# Synthesis and electrochemical performance of NaV_3_O_8_ nanobelts for Li/Na-ion batteries and aqueous zinc-ion batteries

**DOI:** 10.1039/c9ra04339j

**Published:** 2019-07-02

**Authors:** Fang Hu, Di Xie, Fuhan Cui, Dongxu Zhang, Guihong Song

**Affiliations:** School of Materials Science and Engineering, Shenyang University of Technology Shenyang 110870 China hufang25@sut.edu.cn

## Abstract

NaV_3_O_8_ nanobelts were successfully synthesized for Li/Na-ion batteries and rechargeable aqueous zinc-ion batteries (ZIBs) by a facile hydrothermal reaction and subsequent thermal transformation. Compared to the electrochemical performance of LIBs and NIBs, NaV_3_O_8_ nanobelt cathode materials in ZIBs have shown excellent electrochemical performance, including high specific capacity of 421 mA h g^−1^ at 100 mA g^−1^ and good cycle stability with a capacity retention of 94% over 500 cycles at 5 A g^−1^. The good diffusion coefficients and high surface capacity of NaV_3_O_8_ nanobelts in ZIBs were in favor of fast Zn^2+^ intercalation and long-term cycle stability.

## Abstract 2

Compared to the electrochemical performance for LIBs and NIBs, NaV_3_O_8_ nanobelts electrode for ZIBs shows excellent electrochemical performance, including high specific capacity of 421 mA h g^−1^ at 100 mA g^−1^, good rate performance and cycle performance.

## Graphical abstract

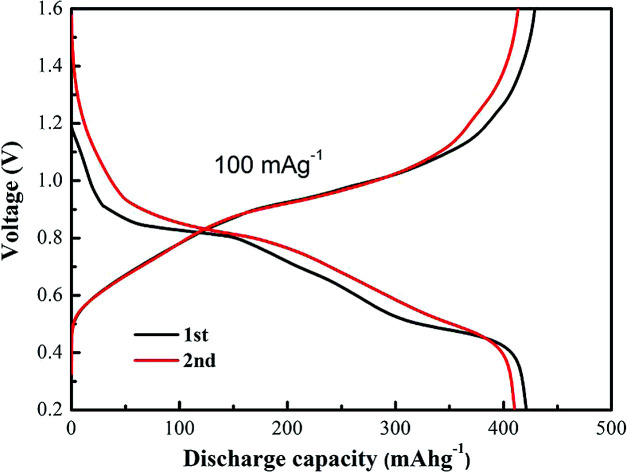

## 1. Introduction

In large-scale electric energy storage applications, lithium-ion batteries (LIBs) have not met the eventual requirements owing to limited lithium resources, safety hazards, high cost and low power density. Sodium-ion batteries have been brought into focus for renewable energy and smart grids because of the abundant sodium resources and low cost.^1–3^ However, the Na^+^ has a larger ionic radius (1.06 Å) than Li^+^ (0.76 Å), which causes sluggish Na^+^ ion kinetics, reversible intercalation difficulties and electrochemical performances lower than those for Li-ion batteries.^4,5^ Recently, the extensive study of aqueous zinc ion batteries (ZIBs) has begun owing to their low cost, high theoretical capacity (820 mA h g^−1^), environmentally friendliness and rich global distribution.^6–8^ The radius of the Zn^2+^ ion (0.74 Å) is almost the same as that of the Li^+^ ion (0.76 Å), but the development of rechargeable aqueous Zn-ion batteries is seriously impeded by the limited choices of suitable cathode materials because of the larger atomic mass and stronger positive polarity.^8^ α-MnO_2_,^9^ β-MnO_2_,^10^ γ-MnO_2_ (ref. 11) and ZnMn_2_O_4_ (ref. 12) have been explored as cathode materials for ZIBs, but the MnO_2_ cathode suffers from poor rate performance and rapid capacity fading due to its inherent low conductivity and the Jahn–Teller distortion effect, while ZnMn_2_O_4_ delivers limited capacity around 90 mA h g^−1^ at a current of 500 mA g^−1^.

In the past, layered vanadium-based compounds have been exhibited good electrochemical performance for LIBs and NIBs because of the multiple oxidation state of vanadium and wide interlayer spacing.^13–17^ Now, layered vanadium-based compounds, such as Zn_0.25_V_2_O_5_·*n*H_2_O,^18^ V_2_O_5_·*n*H_2_O,^19^ H_2_V_3_O_8_,^20^ V_3_O_7_·H_2_O,^21^ Na_0.33_V_2_O_5_,^22^ VS_2_ (ref. 23) and Zn_2_V_2_O_7_,^24^ can also act as promising host materials for ZIBs based on intercalation reaction. Nazar's group reported hydrothermal synthesis of Zn_0.25_V_2_O_5_·*n*H_2_O nanobelts, which exhibited a specific energy of about 250 W h kg^−1^, a capacity of 220 mA h g^−1^ at 4.5 A g^−1^, and a capacity retention of more than 80% after 1000 cycles.^18^ Kim and his coworkers prepared Zn_2_V_2_O_7_ nanowires, which delivered a specific energy of 166 W h kg^−1^ at 50 mA g^−1^. The cycling performance of Zn_2_V_2_O_7_ indicates a 85% of the maximum capacity after 1000 cycles at 4 A g^−1^.^24^ Liang' group obtained Na_1.1_V_3_O_7.9_ nanoribbons/graphene with a high capacity of 220 mA h g^−1^ at 300 mA g^−1^, and a high capacity retention of 77% with respect to the highest value after 100 cycles.^25^

Recently, layered vanadium-based compounds NaV_3_O_8_ has been reported as a cathode material for LIBs, NIBs and ZIBs, respectively. Cao *et al.* synthesized NaV_3_O_8_ nanoplates by using an *in situ* template method. The electrode can deliver a high capacity of 230 mA h g^−1^ at the current density of 100 mA g^−1^ with capacity retention of 93.4% after 200 cycles.^14^ He *et al.* reported hydrothermal synthesis NaV_3_O_8_ nanowires with the raw material of V_2_O_5_ and NaOH, and they present a high discharge capacity of 145.8 mA h g^−1^ at the current density of 10 mA g^−1^ and good retention of 91.1% after 50 cycles for NIBs.^26^ Mai group fabricated NaV_3_O_8_ nanowires by annealing Na_2_V_6_O_16_·1.63H_2_O at 400 °C, which only shows a discharge capacity of 240 mA h g^−1^ at the current density of 50 mA g^−1^ for ZIBs.^27^

Herein, we develop a facile hydrothermal reaction and subsequent thermal transformation to obtain NaV_3_O_8_ nanobelts. The as-prepared NaV_3_O_8_ nanobelts are investigated as the cathode material for aqueous zinc ion batteries, which exhibited superior zinc ions storage performances including large specific capacity (421 mA h g^−1^ at 100 mA g^−1^), high rate capability (120 mA h g^−1^ at 10 A g^−1^) and long-term cycling stability (a retention of 94% at 5 A g^−1^ after 500 cycles).

## 2. Experimental

All chemicals were of analytical grade and were used as received without further purification. In a typical procedure, 0.24 g NaOH, 0.234 g NH_4_VO_3_ and 0.053 g Na_2_CO_3_ were added together to a beaker with 50 mL of distilled water and the solution was stirred at 90 °C for 20 min. Then, put the beaker into cool water to room temperature. 1 mL 30% H_2_O_2_ was dissolved in the solution and stirred about 2 min with the solution color changed to faint yellow. The solution was adjusted to pH = 1.9 by the titration of 2 mol L^−1^ HCl and kept for stirring about 30 min, the solution color turns to reddish brown gradually. Finally, the solution was thrown into a 50 mL autoclave and subjected to hydrothermal treatment at 200 °C for 24 h. And then, the autoclave was naturally cooled down and red precipitates were collected, followed with wash with distilled water and alcohol for several times, and finally dried at 60 °C. At last, the product was also annealed in air atmosphere at 400 °C for 4 h with an increasing rate of 3 °C min^−1^.

The crystallographic structure and phase purity of the as-prepared products were measured by a powder X-ray diffraction analyzer (XRD, 7000, Shimadzu) with Cu Kα radiation (*λ* = 1.5406 Å). The morphology and structure of the samples were characterized by using a scanning electron microscope (SEM, Hitachi-4800) and a high resolution transmission electron microscope (HRTEM, JEM-2100 PLUS) operated at 200 kV. X-ray photoelectron spectra (XPS) measurements were conducted using an ESCALAB250 to investigate the elemental composition using an Al Kα source. The nitrogen adsorption–desorption isotherms at the temperature of liquid nitrogen (77 K) were measured on VSorb 4800P Surface Area and Pore Porosimetry Analyzer (Gold Spectrum Technology Co., Ltd., China) with prior degassing under vacuum for 4 h at 150 °C. Total pore volumes were determined using the adsorbed volume at a relative pressure of 0.99. The multi-point Brunauer–Emmett–Teller (BET) surface area was estimated from the relative pressure range from 0.05 to 0.2.

Galvanostatic discharge/charge measurements were carried out using Land-2100 automatic battery tester (Land-2100, China). Electrochemical tests were performed by using a coin battery cell, where a metallic lithium foil, sodium foil and zinc foil were used as the anode electrode for Li/Na ion batteries and aqueous zinc ion batteries, respectively. For Li/Na coin cells, NaV_3_O_8_ active materials (70%, mass fraction) were blended with acetylene black (20%, mass fraction) and polyvinylidene fluoride (PVDF, 10%, mass fraction). The resultant slurry was pasted on an Al foil as cathode electrode and then dried in a vacuum oven. For zinc coin cells, the cathode electrode was fabricated by pressing a mixture of 70% active material, 20% conductive carbon and 10% poly tetra fluoroethylene (PTFE) onto a carbon paper and dried under air at 60 °C for 12 h. A Whatman grade glass fiber was chosen as separator between cathode and anode for Na ion batteries and aqueous zinc ion batteries, respectively. 1 mol L^−1^ lithium hexafluorophosphate (LiPF_6_) dissolved in ethylene carbonate (EC), dimethyl carbonate (DMC) and ethyl methyl carbonate (EMC) (EC : DMC : EMC = 1 : 1 : 8, by v/v ratio) was used as electrolyte. For Na coin cells, the electrolyte was 1 mol L^−1^ NaClO_4_ dissolved in a solvent mixture of ethylene carbonate (EC) and propylene carbonate (PC) (1 : 1 v/v) and 5% FEC. For zinc ion batteries, the coin cells were assembled under room temperature and 1 mol L^−1^ ZnSO_4_·7H_2_O and 0.2 mol L^−1^ Na_2_SO_4_ mixed solution was used as the electrolyte. Cyclic voltammetry (CV) was carried out using Bio-Logic VSP-300 multichannel electrochemical workstations.

## 3. Results and discussion

**Fig. 1:**
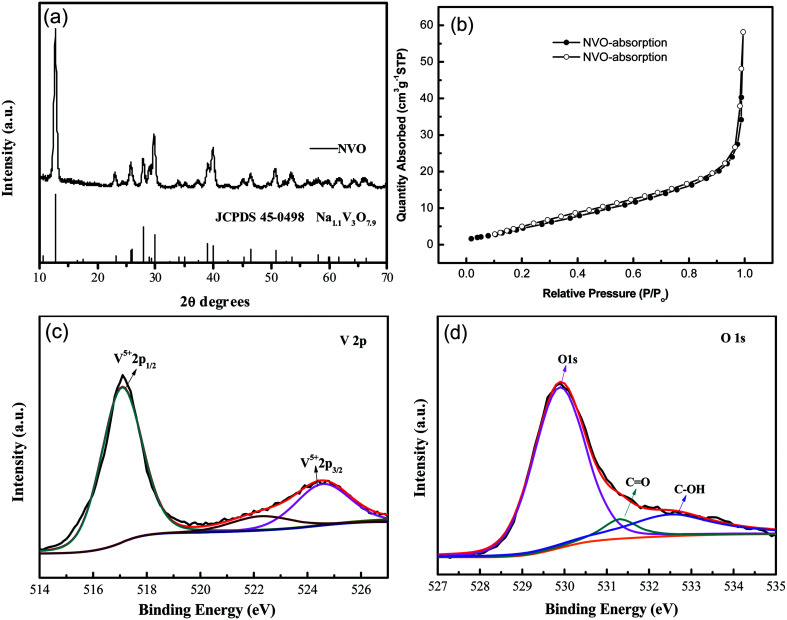
(a) XRD patterns of the as-synthesized products; (b) N_2_ adsorption–desorption isotherm of NVO samples. XPS survey spectrum of NVO samples (c) V 2p and (d) O 1s.

XRD patterns of the as-synthesized NaV_3_O_8_ (hereafter denoted as NVO) products are showed in Fig. 1a. The diffraction peaks of the as-synthesized NVO could be well indexed to Na_1.1_V_3_O_7.9_ phase (JCPDS no. 45-0498), and no other impurities are detected. Nitrogen adsorption–desorption isotherms characterization (Fig. 1b) revealed that the Brunauer–Emmett–Teller (BET) surface area of the as-prepared NVO was 25.5 cm^2^ g^−1^, which could provide large contact area between the materials and electrolyte. XPS analysis of V and O was performed as shown in Fig. 1c and d, respectively. The peaks position of V are centered at 517 eV and 524.5 eV, respectively. The spin–orbit coupling splits the V 2p states into *j* = 3/2 and *j* = 1/2 by about 7.5 eV.^28^ The peaks located at lower energy of 529.8, 531.3, and 532.5 eV could be assigned to O 1s in NVO, which is similar to the that of H_2_V_3_O_8_.^29^ The first one corresponds to O^2−^ ion in the V–O bonding, the second and the third are assigned to OH group in H_2_O molecule and C=O on the surface, respectively.

In our experiment, multi-step synthesis of NaV_3_O_8_ nanobelts can be described by the following reactions:

1 NH_4_^+^ + VO_3_^−^ + 3OH^−^ → VO_4_^3−^ + NH_3_↑ + 2H_2_O

2 VO_4_^3−^ + 2H_2_O_2_ → [VO_2_(O_2_)_2_]^3−^ + 2H_2_O

3 2Na^+^ + 4H^+^ + 4[VO_2_(O_2_)_2_]^3−^ + 2[V(O_2_)]^3+^ + H_2_O → Na_2_V_6_O_16_·3H_2_O + 5O_2_↑

4
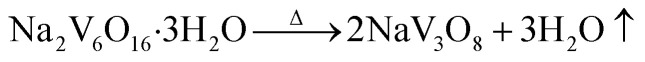


The reaction (2) showed that [VO_2_(O_2_)_2_]^3−^ can be formed by added H_2_O_2_ with the solution color turned to faint yellow. For the third step, when the pH value of the solution decreased to 1.9, some [VO_2_(O_2_)_2_]^3−^ can be converted to [V(O_2_)]^3+^ with the solution changed to reddish brown. As we known, [VO_2_(O_2_)_2_]^3−^ can be converted to [V(O_2_)]^3+^ totally when the pH value is 1.0.

**Fig. 2:**
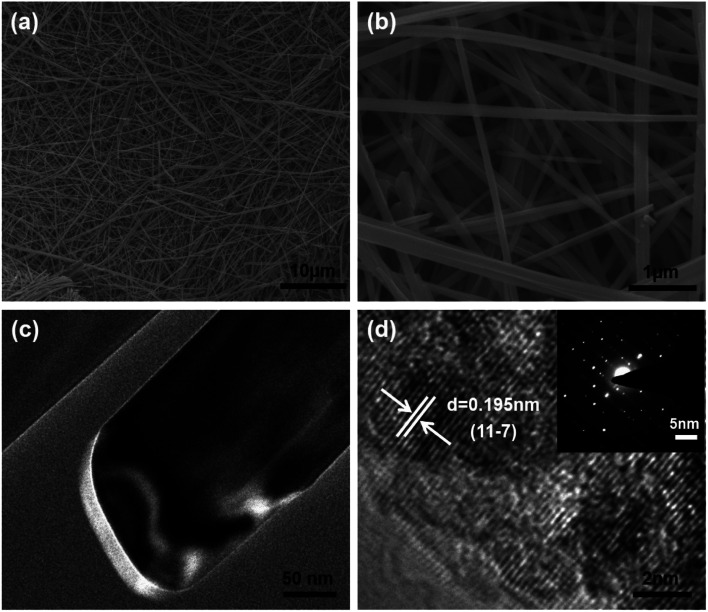
(a) Low magnification SEM images of NVO nanobelts. (b) High magnification SEM images of NVO nanobelts; (c) TEM images of the NVO nanobelts; (d) HRTEM and SAED (inset) image of NVO nanobelts.

The SEM image in Fig. 2a and b shows the NVO is composed of long belts-like with lengths of above 50 μm and widths of about 200–300 nm. The TEM results in Fig. 2c show the thickness of NVO nanobelts are about 30 nm. HRTEM in Fig. 2d shows the lattice fringes of 0.195 nm corresponded to the (11–7) planes of NaV_3_O_8_ and the spot in the SAED pattern was parallel to the length of the NVO nanobelts, indicating the growth direction of the material.

**Fig. 3:**
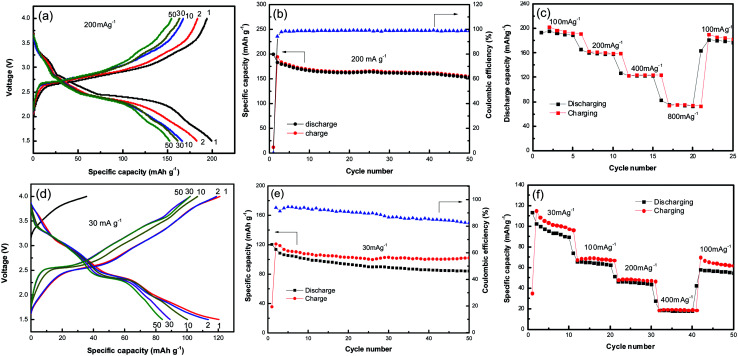
Galvanostatic charge–discharge profiles (a and d) and cycle performance (b and e) of NVO nanobelts at a current density of 200 mA g^−1^ and 30 mA g^−1^ with Li and Na in the voltage range from 4 V to 1.5 V, respectively. (c and f) The rate performance of NVO nanobelts at the different current density with Li and Na, respectively.

Fig. 3a shows the galvanostatic charge–discharge profiles of NVO nanobelts at a current density of 200 mA g^−1^ with Li in the voltage range from 4 V to 1.5 V. It can be observed that the discharge plateaus located at around 3.2, 2.7 and 2.4 V, implying a complex multiphase transition mechanism during the Li-ion insertion process. The initial discharge capacity of NVO nanobelts is about 200 mA h g^−1^, which corresponds to a 2.26 electron transfer reaction. A high charge capacity of 195 mA h g^−1^ can be obtained, indicating a high reversibility of NVO nanobelts cathode materials for Li insertion/extraction reactions. After 50 cycles in Fig. 3b, NVO nanobelts can maintain discharge capacity of 153 mA h g^−1^ with a capacity retention of 76.5% and high coulombic efficiency of 99%. Fig. 3c shows the rate performance of NVO nanobelts with Li at the different current densities of 100 mA g^−1^, 200 mA g^−1^, 400 mA g^−1^ and 800 mA g^−1^, respectively. The NVO nanobelts electrode presents a high discharge capacity of 76 mA h g^−1^ at the current density of 800 mA g^−1^. When the current density finally returned to 100 mA g^−1^, a capacity of 177 mA g^−1^ could be recovered, indicating NVO nanobelts remains stable after high rates. In addition, the electrochemical performance of NVO nanobelts as cathode materials in the voltage range from 4 V to 1.5 V for Na-ion batteries was also investigated (Fig. 3d–f). The initial discharge capacity of NVO nanobelts with Na is about 120 mA h g^−1^ at a current density of 30 mA g^−1^ with two average plateaus at about 3.0 and 2.39 V. The charge–discharge profiles at different cycles are overlap reasonably well, indicating the good structural reversibility of NVO nanobelts upon long cycling. The capacity retention after 50 cycles is 70% with coulombic efficiency of 84%. We speculate that Na ions in NaV_3_O_8_ cannot act as a pillar between the [V_3_O_8_] layers in Na ion batteries, which results to the crystal structure of the sample collapsed during the charge and discharge cycle and indicates a low coulombic efficiency.^30^ The electrode presents a rate capability with reversible average discharge capacity of 94, 65, 46 and 18 mA h g^−1^ at the current densities of 30, 100, 200 and 400 mA g^−1^, respectively. When cycling at 100 mA h g^−1^ again, the discharge capacity remains at 56 mA h g^−1^, which indicates NVO nanobelts cathode remains stable after high rates.

**Fig. 4:**
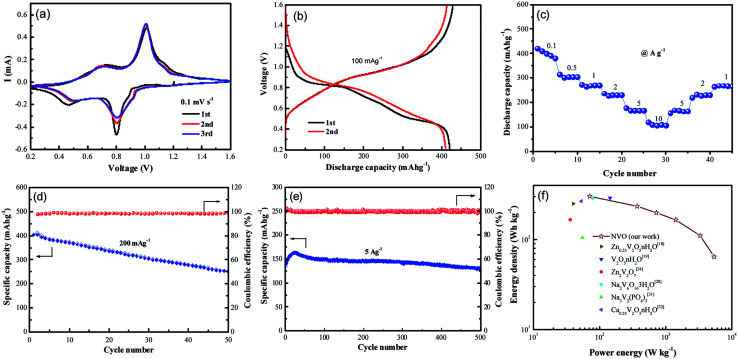
(a) Cyclic voltammograms of NVO electrode at the scan rate of 0.1 mV s^−1^ with the electrochemical window of 0.2–1.6 V for the first three cycles, (b) the discharge/charge curves of NVO at the front two cycles, (c) the rate performance of NVOH at various current densities ranging from 100 mA g^−1^ to 10 A g^−1^. Cycling performance and coulombic efficiency of NVO at the current densities of 200 mA g^−1^ (d) and 5 A g^−1^ (e), respectively. (f) Ragone plots of NVO and other electrodes for ZIBs.

Recently, vanadium-based materials have received extensively attention as cathode for aqueous ZIBs with excellent electrochemical performance. Herein, the electrochemical performance of NVO nanobelts was also investigated. The first three cycled electrochemical Zn-ion storage performance of NVO electrode in Fig. 4a is assessed using CV at 0.1 mV s^−1^ scan rate in the potential range 0.2–1.6 V. Two strong redox peaks on both cathodic and anodic sweeps are observed, corresponding to continuous reduction (Zn^2+^ insertion) and oxidation (Zn^2+^ extraction) of NVO electrode. The redox couple at about 0.80/1.01 V in the cathodic/anodic sweep represents the redox couple at the equilibrium voltage V^5+^/V^4+^ in NVO electrode. Similarly, the redox couple at 0.46/0.73 V represents the V^4+^/V^3+^ couple. The multiphase vanadium redox couples commonly induce multistep electrochemical intercalation/extraction of various charge carriers in the vanadium-based layered structure. The second and third discharge profiles are similar and slightly different from the first, indicating a stead structure after first cycle. The largely overlapped CV curves for the NVO electrode suggest the good reversibility of the zinc ions interaction/extraction process and the good retention of crystallinity.

Galvanostatic first charge–discharge profiles of NVO electrodes with Zn are examined at a current density of 100 mA g^−1^ in the voltage range from 0.2 V to 1.6 V (Fig. 4b). In agreement with the CV curves, the NVO electrode exhibits a profile with two discharge plateau at about 0.84 V and 0.5 V during discharge, respectively. The NVO electrode delivers initial specific discharge capacity of 421 mA h g^−1^, which is close to the theoretical capacity 529 mA h g^−1^ of NVO corresponding to 2.37 mol Zn^2+^ insertion. The calculated first charge capacity is 428 mA h g^−1^ with a high initial coulombic efficiency (101.7%) for NVO. In the second and third discharge process, these two voltage plateaus of NVO remained in every profile. This phenomenon suggested that the electrochemical reactions might occur without irreversible crystal structure change in the subsequent cycles. The rate performance of NVO electrodes is also tested in Fig. 4c. The average discharge capacities are 400, 303, 270, 231, 166 and 105 mA h g^−1^ at current densities of 0.1, 0.5, 1, 2, 5 and 10 A g^−1^, respectively. When the rate is returned to 1 A g^−1^, the electrode is able to recover a reversible capacity of 270 mA h g^−1^, indicating a high reversibility of NVO nanobelts cathode materials for Zn insertion/extraction reactions.

Fig. 4d shows the cycling performance and coulombic efficiency of the NVO electrodes at the current density of 200 mA g^−1^, respectively. After 50 cycles, the discharge capacities are reduced from 400 to 253 mA h g^−1^, corresponding to the capacity retention of 62%. Fig. 4e shows the cycling performance of NVO in large current density of 5 A g^−1^. The increase specific capacity of NVO from 139 mA h g^−1^ to the maximum capacity of 163 mA h g^−1^ for about the initial 20 cycles is attributed to the activation. After reaching a steady state, the electrode exhibits a decrease in capacity, and at the end of 500 cycles, achieves a capacity of 131 mA h g^−1^ with retention of 94% of the first discharge capacity, indicating the outstanding rate ability and stable cycling ability of NVO nanobelts electrode.

The Ragone plot in Fig. 4f can be used to evaluate the practical suitability of the NVO electrode along with reported cathode for ARZIBs. This electrode delivers a high specific energy of 302 W h kg^−1^ at the specific power of 71 W kg^−1^ based on the active mass of the cathode material, which surpasses those cathodes such as Zn_0.25_V_2_O_5_·*n*H_2_O,^18^ V_2_O_5_·*n*H_2_O,^19^ Zn_2_V_2_O_7_,^24^ Na_2_V_6_O_16_·3H_2_O,^28^ Na_3_V_2_(PO_4_)_3_ (ref. 31) and Ca_0.25_V_2_O_5_·*n*H_2_O.^32^ The energy density still remained at a high value of 64 W h kg^−1^ when the power density was as high as 5440 W kg^−1^, indicating a fast and good electrochemical performance of NVO electrode for ARZIBs.

**Fig. 5:**
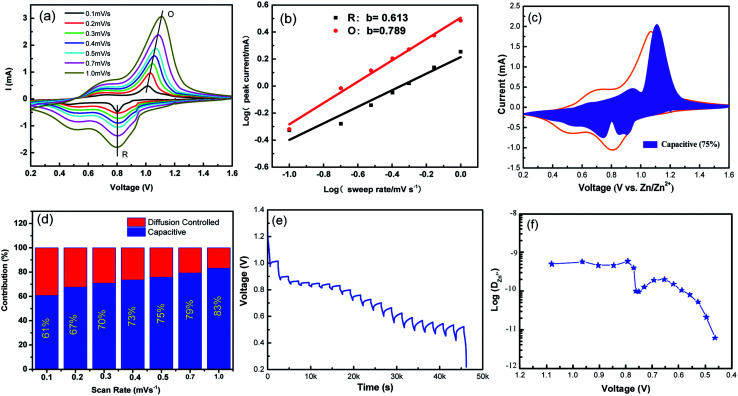
(a) Cyclic voltammetry curves of the NVO electrodes at different voltage scan rates varies from 0.1 to 1.0 mV s^−1^ for aqueous Zn-ion batteries. (b) log(*i*)–log(*ν*) plots for specific peak currents at different scan rates to analysis the diffusion/capacity behaviors. (c) The contribution ratio of the capacitive capacities and diffusion-limited capacities based on eqn (2) at 0.5 mV s^−1^. (d) Bar diagram for contribution ratio between capacitive capacities and diffusion-limited capacities for different voltage scan rates. (e) GITT data collected at a current density of 100 mA g^−1^ for 10 min and a rest interval of 30 min. (f) The corresponding ionic diffusion coefficient.

To further understand the electrochemical kinetics, cyclic voltammetry curves of the NVO electrodes at different voltage scan rates varies from 0.1 to 1.0 mV s^−1^ with Zn were shown in Fig. 5a. The electrochemical kinetics process can be assumed to fit the following eqn (5).^33^

5 *i* = *aν*^*b*^

where *i* and *ν* are the peak current (mA) and the corresponding scan rate (V s^−1^), respectively; *a* and *b* are variable parameters in which the latter varies in the range of 0.5–1.0. For a given system, the *b* value of 0.5 depicts the process of diffusion-limited and the value is 1.0 indicating a capacitive process. Based on the relationship between log(*i*) and log(*ν*) for two different peaks, the *b*-values determined by the slopes of the four redox peaks are 0.61 and 0.79 (Fig. 5b), inferring that the electrochemical kinetics regulation are diffusion-influenced as shown in Fig. 5c.

From eqn (5), to further analyze the combination of surface-controlled capacitive and diffusion-controlled intercalation processes, the following equation is derived.^34^

6 *i* = *k*_1_*v* + *k*_2_*v*^1/2^

where the first and second compartments in the right side represent the capacity- and diffusion-limited redox reactions, respectively. The calculated capacity- and diffusion-controlled redox values at different scan rates (0.1, 0.2, 0.3, 0.4, 0.5 0.7 and 1 mV s^−1^) are calculated as given in Fig. 5c and d. At the scan rate of 0.1 mV s^−1^, 61% of the capacitive contribution is capacity-limited. When the scan rate improved to 1 mV s^−1^, nearly 83% of the capacitive contribution is capacity-limited. The results indicate that the capacitance effect play an increasingly important role as the scanning rate is increased. The electrochemical kinetics of NVO indicates that the surface dominated capacitive behaviors of extrinsic NVO nanobelts are in favor of fast Zn^2+^ intercalation/extraction.

In addition, to determining the average diffusion coefficient around the redox peaks, galvanostatic intermittent titration technique (GITT) measurements are carried out to better understand the kinetics during the diffusion of Zn^2+^ ions in NVO. The GITT data were collected at a current density of 100 mA g^−1^ for 10 min and a rest interval of 30 min (Fig. 5e). The ionic diffusion coefficient in NVO electrodes as a function of voltage can be determined by the following equation.^35^7
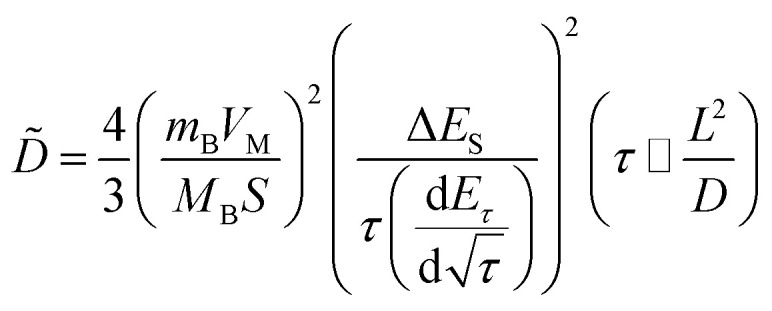
where *V*_M_, *M*_B_, *m*_B_, and *S* are the molar volume, the molecular weight of the material and the mass, the active surface area of the electrode, respectively. *L* is the average thickness of the electrode. If the cell voltage is linearly proportional to *τ*^1/2^, eqn (8) can be further simplified to the following^36^8
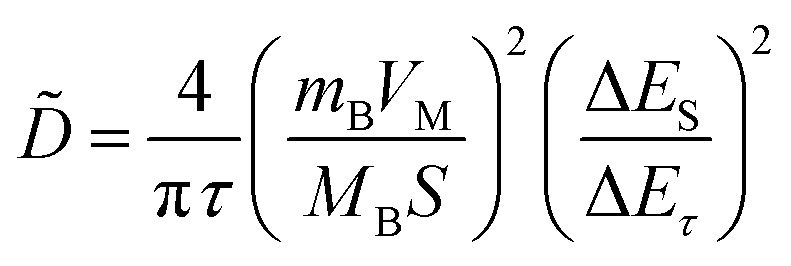
where Δ*E*_*τ*_ and Δ*E*_S_ represent the total change in the cell voltage during the current pulse and the change in the steady-state voltage of the cell during the step at the plateau potential, respectively. On this basis, one can calculate Zn ions diffusion coefficients of NVO is about 5.0 × 10^−10^ cm^2^ s^−1^ to 6.2 × 10^−12^ cm^2^ s^−1^ (Fig. 5f). The diffusion coefficients are found to strongly depend on the electrochemical process. The high Zn ions diffusion coefficients would be responsible for the remarkable electrochemical performance of NVO.

**Fig. 6:**
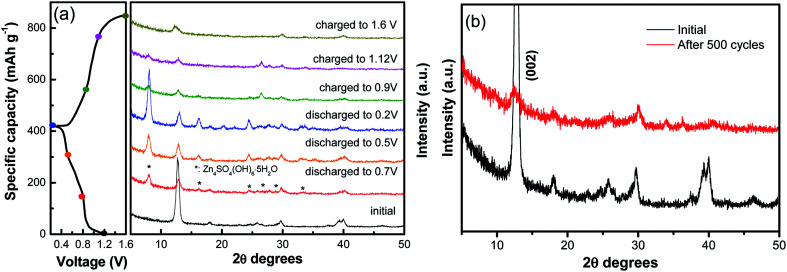
(a) *Ex situ* XRD analysis of the NVO nanobelts during the first charge/discharge cycle at the current density of 100 mA g^−1^. (b) XRD patterns of the electrode after 500 cycles at a current density of 5 A g^−1^.

Furthermore, *ex situ* XRD analysis in Fig. 6 was carried out to investigate the structure evolution of the NVO nanobelts during the first charge/discharge cycle at the current density of 100 mA g^−1^. The obvious reflection peak at 2*θ* = 12.7° (*d* = 0.69 nm) as attributed to the (002) plane of NVO. When the electrode was discharged to 0.8 V, a few new peaks appeared at 8.0°, 16.1°, 24.5°, 26.6°, 28.7° and 32.9°, which could be indexed to a triclinic Zn_4_SO_4_(OH)_6_·5H_2_O phase (JCPDS no. 39-0688). The (002) plane of NVO becomes weaker and slightly shifted toward higher 2*θ* positions from 12.7° to 12.9°. With more Zn^2+^ intercalated into the electrode from 0.8 V to 0.2 V, the reflection peaks of Zn_4_SO_4_(OH)_6_·5H_2_O are gradually strengthened, and the (002) plane of NVO continuously shifted to a higher 2*θ* position (13.0°), which corresponded to an enlarged interlayer distance of 0.67 nm. This reduction in the interplanar spacing from 0.69 to 0.67 nm indicates an improvement in the structural coordination owing to the strong electrostatic interaction between the intercalated Zn^2+^ and the (V_3_O_8_)^−^ layers.^28^ During the subsequent charging process, the reflection peaks of Zn_4_SO_4_(OH)_6_·5H_2_O gradually disappeared. The formation of this phase can be described by the following reactions:^37^

9 3Zn + 6OH^−^ + Zn_4_SO_4_·*x*H_2_O + (5 − *x*)H_2_O − 6e^−^ ↔ Zn_4_SO_4_(OH)_6_·5H_2_O

Meanwhile, the (002) plane of NVO shifted back to a lower 2*θ* position from 13.0° to 12.5°, which demonstrated that the Zn^2+^ intercalation and extraction in NVO was highly reversible. The XRD of the electrode after 500 cycles at a current density of 5 A g^−1^ is investigated in Fig. 6b. The main diffraction peak corresponding to the (002) plane still remains after 500 cycles, indicating a stable structure of the sample and a highly reversible zinc ion intercalation/de-intercalation process in NaV_3_O_8_ electrode.

## 4. Conclusion

In this work, a facile hydrothermal reaction was developed to prepare NaV_3_O_8_ nanobelts as a promising cathode material for LIBs, NIBs and ZIBs, respectively. Compared to the electrochemical performance for LIBs and NIBs, NaV_3_O_8_ nanobelts cathode materials for ZIBs have shown excellent electrochemical performance, including high specific capacity of 421 mA h g^−1^ at 100 mA g^−1^ and good cycle stability with a capacity retention of 94% over 500 cycles at 5 A g^−1^. *Ex situ* XRD showed that the Zn^2+^ intercalation and extraction in NaV_3_O_8_ nanobelts was highly reversible. The capacitive behavior and good Zn ions diffusion coefficients played an important role in fast Zn^2+^ intercalation/extraction and long cycle stability. This work also indicates that NaV_3_O_8_ nanobelts have a great potential as a cathode material for ZIBs in large-scale electric energy storage applications.

## Conflicts of interest

There are no conflicts to declare.

## Supplementary Material

## References

[cit1] Vaalma C., Buchholz D., Weil M., Passerini S. (2018). Nat. Rev. Mater..

[cit2] Wang D. X., Bie X. F., Fu Q., Dixon D., Bramnik N., Hu Y. S., Fauth F., Wei Y. J., Ehrenberg H., Chen G., Du F. (2017). Nat. Commun..

[cit3] Xie X. Q., Kretschmer K., Anasori B., Sun B., Wang G. X., Gogotsi Y. (2018). ACS Appl. Mater. Interfaces.

[cit4] Hu F., Jiang W., Dong Y. D., Lai X. Y., Xiao L., Wu X. (2017). RSC Adv..

[cit5] Zhao Y. Y., Pang Q., Meng Y., Gao Y., Wang C. Z., Liu B. B., Wei Y. J., Du F., Chen G. (2017). Chem.–Eur. J..

[cit6] Hu E. Y., Yang X. Q. (2018). Nat. Mater..

[cit7] Wang F., Borodin O., Gao T., Fan X. L., Sun W., Han F. D., Faraone A., Dura J. A., Xu K., Wang C. S. (2018). Nat. Mater..

[cit8] Wan F., Zhang L. L., Dai X., Wang X. Y., Niu Z. Q., Chen J. (2018). Nat. Commun..

[cit9] Alfaruqi M. H., Islam S., Gim J., Song J. J., Kim S. J., Pham D. T., Jo J., Xiu Z. L., Mathew V., Kim J. (2016). Chem. Phys. Lett..

[cit10] Zhang N., Cheng F. Y., Liu J. X., Wang L. B., Long X. H., Liu X. S., Li F. J., Chen J. (2017). Nat. Commun..

[cit11] Alfaruqi M. H., Mathew V., Gim J., Kim S., Song J., Baboo J. P., Choi S. H., Kim J. (2015). Chem. Mater..

[cit12] Zhang N., Cheng F. Y., Liu Y. C., Zhao Q., Lei K. X., Chen C. C., Liu X. S., Chen J. (2016). J. Am. Chem. Soc..

[cit13] Zhu L. M., Li W. X., Xie L. L., Yang Q., Cao X. Y. (2019). Chem. Eng. J..

[cit14] Cao L. F., Chen L., Huang Z., Kuang Y. F., Zhou H. H., Chen Z. X. (2016). ChemElectroChem.

[cit15] Chen Z. X., Xu F., Cao S. N., Li Z. F., Yang H. X., Ai X. P., Cao Y. L. (2017). Small.

[cit16] Liang S. Q., Zhou J., Fang G. Z., Zhang C., Wu J., Tang Y., Pan A. Q. (2014). Electrochim. Acta.

[cit17] Kim J. K., Senthilkumar B., Sun H. S., Kim J. H., Chi M. F., Kim Y. (2015). ACS Appl. Mater. Interfaces.

[cit18] Kundu D., Adams B. D., Duffort V., Vajargah S. H., Nazar L. F. (2016). Nat. Energy.

[cit19] Yan M. Y., He P., Chen Y., Wang S. Y., Wei Q. L., Zhao K. N., Xu X., An Q. Y., Shuang Y., Shao Y. Y., Mueller K. T., Mai L. Q., Liu J., Yang J. H. (2018). Adv. Mater..

[cit20] He P., Quan Y. L., Xu X., Yan M. Y., Yang W., An Q. Y., He L., Mai L. Q. (2017). Small.

[cit21] Kundu D., Vajargah S. H., Wan L. W., Adams B., Prendergastbc D., Nazar L. F. (2018). Energy Environ. Sci..

[cit22] He P., Zhang G. B., Liao X. B., Yan M. Y., Xu X., An Q. Y., Liu J., Mai L. Q. (2018). Adv. Energy Mater..

[cit23] He P., Yan M. Y., Zhang G. B., Sun R. M., Chen L. N., An Q. Y., Mai L. Q. (2017). Adv. Energy Mater..

[cit24] Sambandam B., Soundharrajan V., Kim S., Alfaruqi M. H., Jo J., Kim S., Mathew V., Sun Y. K., Kim J. (2018). J. Mater. Chem. A.

[cit25] Cai Y. S., Liu F., Luo Z. G., Fang G. Z., Zhou J., Pan A. Q., Liang S. Q. (2018). Energy Storage Materials.

[cit26] He H. N., Jin G. H., Wang H. Y., Huang X. B., Chen Z. H., Sun D., Tang Y. G. (2014). J. Mater. Chem. A.

[cit27] Hu P., Zhu T., Wang X. P., Wei X. J., Yan M. Y., Li J. T., Luo W., Yang W., Zhang W. C., Zhou L., Zhou Z. Q., Mai L. Q. (2018). Nano Lett..

[cit28] Soundharrajan V., Sambandam B., Kim S., Alfaruqi M. H., Putro D. Y., Jo J., Kim S., Mathew V., Sun Y. K., Kim J. (2018). Nano Lett..

[cit29] Pang Q., Sun C. L., Yu Y. H., Zhao K. N., Zhang Z. Y., Voyles P. M., Chen G., Wei Y. J., Wang X. D. (2018). Adv. Energy Mater..

[cit30] Hu F., Jiang W., Dong Y. D., Lai X. Y., Xiao L., Wu X. (2017). RSC Adv..

[cit31] Li G. L., Yang Z., Jiang Y., Jin C. H., Huang W., Ding X. L., Huang Y. H. (2016). Nano Energy.

[cit32] Xia C., Guo J., Li P., Zhang X. X., Alshareef H. N. (2018). Angew. Chem., Int. Ed..

[cit33] Wang D., Wei Q., Sheng J., Hu P., Yan M., Sun R., Xu X., An Q. Y., Mai L. Q. (2016). Phys. Chem. Chem. Phys..

[cit34] Wang J., Polleux J., Lim A. J., Dunn B. (2017). J. Phys. Chem. C.

[cit35] Weppner W., Huggins R. A. (1977). J. Electrochem. Soc..

[cit36] Rui X. H., Ding N., Liu J., Li C., Chen C. H. (2010). Electrochim. Acta.

[cit37] Chen G. P., Sang S. B., Huang K. L., Tang Y. G., Tang Z. Q., Yang Z. H. (2004). Trans. Nonferrous Met. Soc. China.

